# The essential genome of *Streptococcus agalactiae*

**DOI:** 10.1186/s12864-016-2741-z

**Published:** 2016-05-26

**Authors:** Thomas A. Hooven, Andrew J. Catomeris, Leor H. Akabas, Tara M. Randis, Duncan J. Maskell, Sarah E. Peters, Sandra Ott, Ivette Santana-Cruz, Luke J. Tallon, Hervé Tettelin, Adam J. Ratner

**Affiliations:** Department of Pediatrics, Columbia University, New York, NY USA; Department of Pediatrics, Division of Pediatric Infectious Diseases, New York University School of Medicine, 550 First Avenue (MSB 223), New York, NY 10016 USA; Department of Microbiology, New York University School of Medicine, New York, NY USA; Department of Veterinary Medicine, University of Cambridge, Cambridge, UK; Institute for Genome Sciences, University of Maryland School of Medicine, Baltimore, MD USA

## Abstract

**Background:**

Next-generation sequencing of transposon-genome junctions from a saturated bacterial mutant library (Tn-seq) is a powerful tool that permits genome-wide determination of the contribution of genes to fitness of the organism under a wide range of experimental conditions. We report development, testing, and results from a Tn-seq system for use in *Streptococcus agalactiae* (group B *Streptococcus*; GBS), an important cause of neonatal sepsis.

**Methods:**

Our method uses a *Himar1* mini-transposon that inserts at genomic TA dinucleotide sites, delivered to GBS on a temperature-sensitive plasmid that is subsequently cured from the bacterial population. In order to establish the GBS essential genome, we performed Tn-seq on DNA collected from three independent mutant libraries—with at least 135,000 mutants per library—at serial 24 h time points after outgrowth in rich media.

**Results:**

After statistical analysis of transposon insertion density and distribution, we identified 13.5 % of genes as essential and 1.2 % as critical, with high levels of reproducibility. Essential and critical genes are enriched for fundamental cellular housekeeping functions, such as acyl-tRNA biosynthesis, nucleotide metabolism, and glycolysis. We further validated our system by comparing fitness assignments of homologous genes in GBS and a close bacterial relative, *Streptococcus pyogenes*, which demonstrated 93 % concordance. Finally, we used our fitness assignments to identify signal transduction pathway components predicted to be essential or critical in GBS.

**Conclusions:**

We believe that our baseline fitness assignments will be a valuable tool for GBS researchers and that our system has the potential to reveal key pathogenesis gene networks and potential therapeutic/preventative targets.

**Electronic supplementary material:**

The online version of this article (doi:10.1186/s12864-016-2741-z) contains supplementary material, which is available to authorized users.

## Importance

Despite the current prevention strategy of administering prophylactic antibiotics to colonized pregnant women, GBS remains a major cause of morbidity and mortality among neonates. New, targeted approaches to treatment and prevention of GBS infection in this population are needed. The assignment of genome-wide fitness categories can illuminate critical molecular networks and can help identify candidate targets for novel antimicrobials. We have developed and tested a Tn-seq system for use in GBS, which we used to generate genome-wide fitness assignments. In addition to providing important baseline data about fundamental GBS biology, our Tn-seq system represents a potentially valuable tool for identifying conditionally essential genes in any of the many extant models of GBS infection.

## Background

The number of publicly available bacterial whole genome sequences is rising exponentially [1], creating a wealth of information about bacterial genetic diversity. Less is known about the extent to which individual genes and gene networks contribute to bacterial fitness in a given environment—information that is valuable for understanding pathogenesis and assessing potential drug targets. Next-generation sequencing (NGS) of transposon-genome junctions from a saturated transposon mutant library (Tn-seq) allows unbiased, genome-wide measurement of the contribution to fitness of individual genes across a wide range of bacterial growth conditions, making it possible to identify essential and conditionally essential genes [2, 3].

*Streptococcus agalactiae* (Group B *Streptococcus*; GBS) is a Gram-positive bacterial species that causes serious neonatal infections, including sepsis, pneumonia, and meningitis [4]. Vertical transmission of GBS to the fetus/newborn can occur *in utero* in the setting of chorioamnionitis (infection of the placenta, fetal membranes, and amniotic fluid), during parturition, or postpartum [4]. Asymptomatic colonization of the gastrointestinal tract and/or vagina occurs in 15-30 % of healthy adults [5–7]. Invasive disease in immunocompetent, non-pregnant, non-elderly adults has been historically rare [8], although several recent reports describe a concerning rise in incidence [9, 10].

The current strategy to prevent neonatal GBS infection is to screen pregnant women for rectovaginal colonization during the third trimester and to administer intrapartum antibiotics to those women with positive screening results [11]. This universal screening approach has dramatically reduced the incidence of early-onset neonatal GBS infection (within the first seven days of life), but has had negligible impact on late-onset GBS infection [12]. Furthermore, the current prevention approach does not aid premature babies born with established GBS infection stemming from vertical transmission *in utero*. These infants are often acutely ill, with multiple organ system dysfunction and poor outcomes [13]. Improved understanding of GBS pathogenesis may allow development of refined prevention/treatment strategies.

While numerous GBS virulence factors and regulatory mechanisms have been identified [14–19], the relative contribution of specific GBS genes and interactions between genes during bacterial colonization and invasion remains incompletely defined. Here we report the essential genome of GBS strain A909, as determined by Tn-seq. We developed and validated a vector-based transposon delivery system that we used to generate multiple unbiased mutant libraries. After statistical analysis of genome insertion sites and determination of the essential genome, genes required for growth in rich media were mapped onto biochemical and signal transduction pathways. This work represents an important step in understanding fundamental molecular interactions required for GBS survival, and provides a baseline dataset against which conditionally essential genes required for pathogenesis can be determined.

## Results

### The modified pCAM48 *Himar1* mini-transposon inserts at random TA sites throughout the GBS genome

**Fig. 1:**
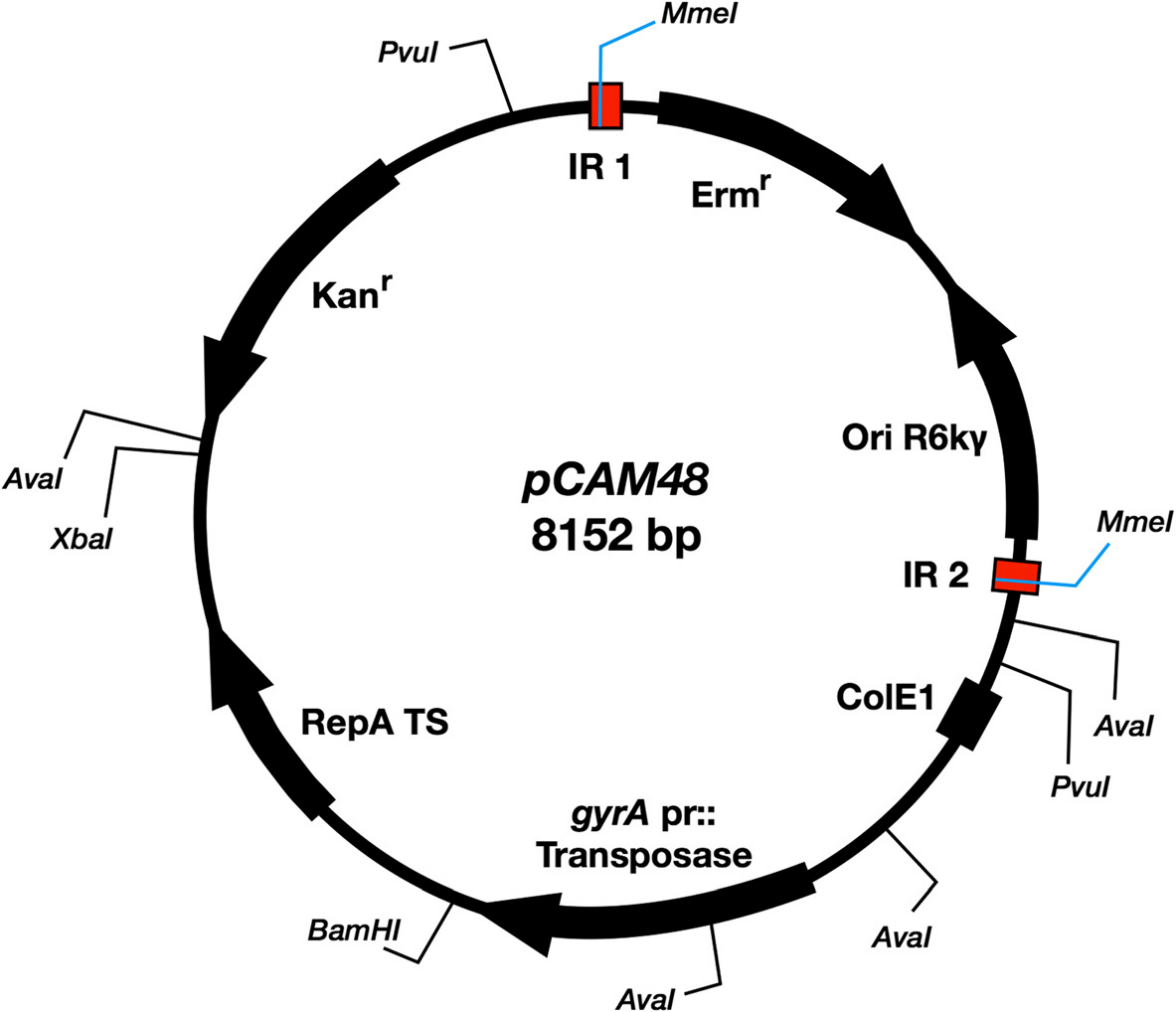
pCAM48 map. pCAM48 is a temperature-sensitive shuttle vector for delivery of a *Himar1* mini-transposon, flanked by inverted repeat (IR) sequences modified to contain MmeI restriction enzyme sites. An erythromycin resistance marker (Erm^r^) is included within the transposon, as is the R6kγ origin of replication (Ori R6kγ), which can be used for plasmid rescue (not employed in this study). Outside of the mini-transposon, the vector contains a ColE1 origin of replication, the Gram-positive temperature-sensitive replicase RepA TS, in which the single-bp deletion present in pCAM45 has been repaired, and a kanamycin resistance marker (Kan^r^). The C9 *Himar1* transposase gene is under the control of the *S. pyogenes* M1 gyrA promoter. Restriction enzyme sites used in development and analysis of pCAM48 are labeled

Our GBS mutant libraries were constructed using a *Himar1* mini-transposon previously used to generate transposon mutant libraries in GBS and related streptococcal species [20, 21]. Past studies that used *Himar1*-based GBS transposon mutant libraries reported Southern blot analysis demonstrating random transposon integration [21]. We developed pCAM48, a temperature-sensitive shuttle vector with an erythromycin (Erm) resistance marker within the mini-transposon that had been modified to contain the MmeI sites required for Tn-seq (Fig. 1). Additionally, pCAM48 was modified from its plasmid progenitor, pCAM45, to correct a single base-pair deletion in the temperature-sensitive origin of replication that prevented proper function [20]. Before subjecting a pCAM48-derived GBS A909 mutant library to NGS analysis in a Tn-seq experiment, we performed multiple quality-control steps in order to ensure that transposition had occurred as expected, that the library had been sufficiently cured of pCAM48, and there was widespread and random integration of our modified mini-transposon throughout the GBS genome.

The first quality control steps involved identification of and selection for clones with the expected antibiotic resistance phenotypes suggesting a) successful transformation with pCAM48 at 28 °C; b) selection for low-frequency transposition events at 37 °C; and c) successful curing of the plasmid after passage at 37 °C. After initial transformation of GBS A909 with pCAM48 and colony growth at the permissive plasmid replication temperature (28 °C), 18–23 individual colonies were patched onto plates with either Erm or Kanamycin (Km) selection, at both the permissive (28 °C) and non-permissive (37 °C) temperatures. Serial dilutions of candidate stocks with the correct antibiotic resistance phenotype (resistant to Erm and Km at 28 °C, Erm resistant but Km sensitive at 37 °C) were plated on tryptic soy (TS) Erm media at 37 °C and TS Erm + Km at 28 °C in order to determine the frequency of transposition, which was between 10^−4^ and 10^−6^ in all tested libraries.

In a second quality control step, intended to insure that the eventual library stock used for Tn-seq had widespread, random transposon insertion mutations with a low rate of identical insertions, 20 individual colonies from each of three candidate libraries were used to seed TS Erm liquid cultures at 37 °C, from which genomic DNA was isolated and subjected to arbitrary priming PCR (APPCR). In each candidate library, APPCR demonstrated uniform transposon dispersion throughout the A909 genome and no siblings. One mutant was revealed by APPCR to have a transposon insertion in the *cylE* gene, whose function is required for production of the hemolytic pigment β-hemolysin/cytolysin [14]. This *cylE* knockout strain was used to confirm the APPCR results using standard PCR with primers specific to the transposon and adjacent genomic DNA. This strain also had the expected non-pigmented phenotype when grown in pigment-enhancing new Granada media (Additional file 1: Figure S1) [22].

### Tn-seq performed on biological triplicate and technical replicate libraries show reliable and reproducible genome-wide transposon insertions

We performed Tn-seq with three mutant libraries that had passed the quality control steps outlined above, which were labeled A2, A5, and A7. Previous Tn-seq studies using similar vector-based transposon delivery systems have reported undesirable persistence of the vector within mutant libraries, which can then be amplified during PCR of transposon-genome junctions, leading to high rates of uninformative sequencing reads that map onto the delivery vector [23]. In order to minimize this outcome, we subjected our libraries to an additional round of plasmid curing by plating our library stocks on 75–80 150 mm × 15 mm TS Erm plates, growing approximately 1x10^6^ colonies for 48 h at 37 °C, then scraping those colonies from the plate to generate final glycerol library stocks. We ultimately observed low rates of vector sequence persistence (between 1.8 and 15.4 % of reads), which is at or below rates observed in other Tn-seq studies [23, 24].

In order to establish the feasibility of performing Tn-seq with our newly developed system, we first performed a pilot experiment with the A2 library. We collected genomic DNA at four time points (T_0_-T_3_) before and after A2 library growth in TS Erm broth at 37 °C. After performing PCR amplification of transposon-genomic junctions, we undertook a limited sequencing run, generating between 8,995,746 and 12,740,228 paired-end reads per condition. Trimming and aligning these reads to the A909 genome revealed, as expected, 17-nucleotide GBS-specific sequences that aligned exclusively to A909 genomic TA sites. However, our measured library saturation (percent of unique TA sites with insertions) was between 5 and 7 % for T_0_-T_2_. Furthermore, at the T_3_ time point, there was an approximately 50 % drop in saturation, similar to a finding reported by Le Breton et al. in their work with *Streptococcus pyogenes* [23].

Following our pilot experiment, we proceeded with growth and Tn-seq analysis of our three libraries, employing more sequencing reads in order to approach full detection of our libraries’ transposon saturation. As in the pilot experiment, genomic DNA samples were collected prior to seeding TS Erm broth at 37 °C (T_0_), and at sequential 24-h time points thereafter. We decided to eliminate the T_3_ time point, however, and excluded the T_3_ pilot data from all future analyses.

**Fig. 2:**
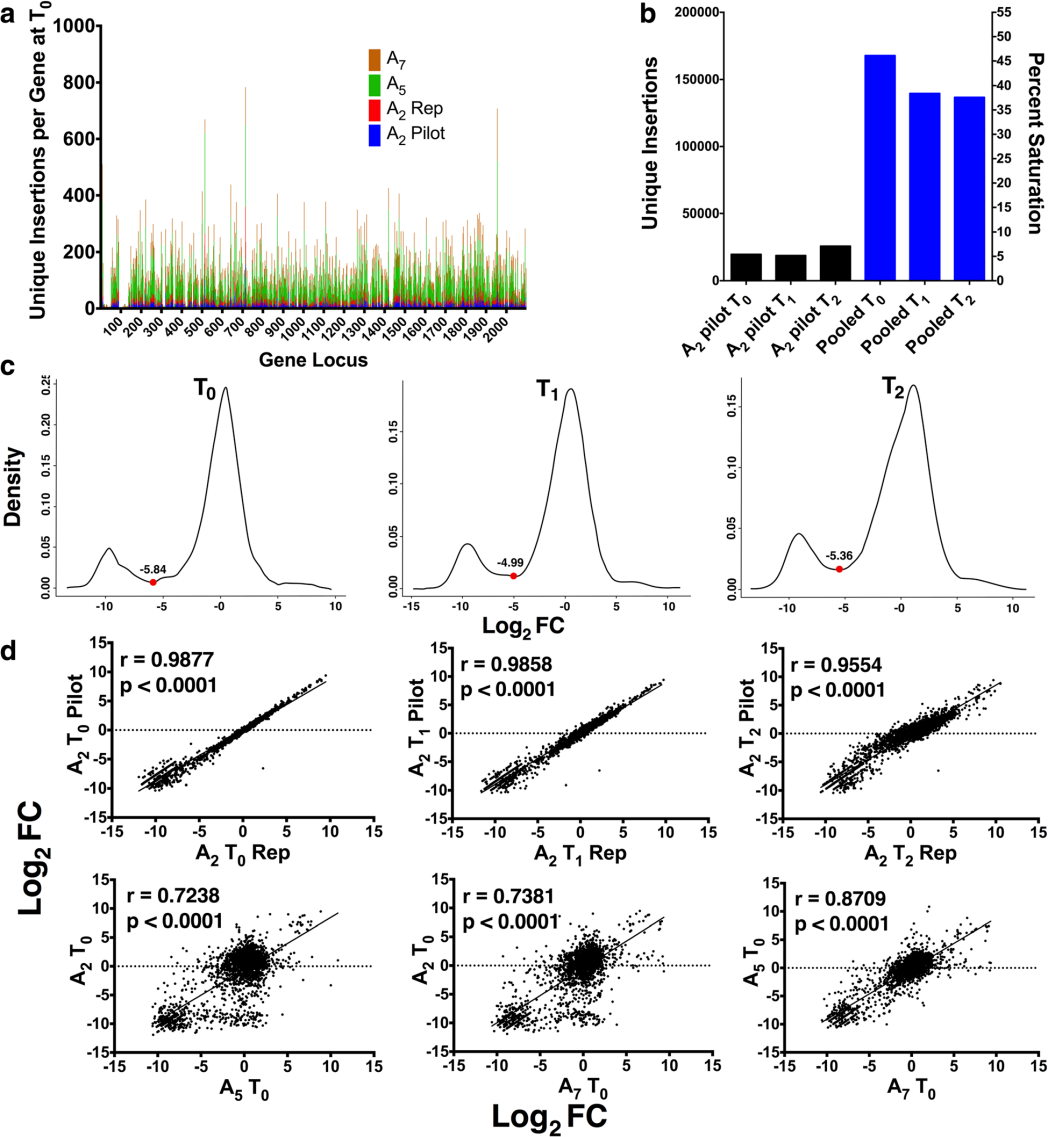
Transposon mutant library metrics. Sequential pooling of data from four T_0_ sequencing runs (for libraries A_2_ pilot, A_2_ repeat, A_5_, and A_7_) demonstrates increasing unique insertion counts per gene (**a**). Library transposon saturation rates (percent of unique TA sites with an insertion) are shown for pilot and pooled datasets (**b**). ESSENTIALS plots of a kernel density function of log transformed measured vs. expected transposon insertion ratios show the expected bimodal distribution separating essential from nonessential genes using pooled libraries. Local minima values were used as determinants of gene essentiality at each time point (**c**). Correlation of library A_2_ log_2_ FC values for each gene from the pilot and technical replicate experiments for each time point, and between the three pairs of biological triplicate libraries for T_0_. Pearson r values and two-tailed *P* values are listed (**d**)

Our experimental design generated, in effect, a pair of technical replicates (the data from the A2 library during the pilot and second experiments) and three pairs of biological triplicates (data from the A2, A5, and A7 libraries). The ESSENTIALS software package compares experimental transposon insertions to the expected number of insertions (based on number of potential insertion sites, sequencing depth, and size of the mutant library) for each gene, and reports the relationship as a logarithmic function of the experimental vs. expected insertion fold-change (log_2_ FC) [25]. There was significant correlation between the log_2_ FC values of our technical and biological replicates (Fig. 2), and the distribution of log_2_ FC values was, as expected, bimodal for all conditions, reflecting the lack of transposon insertions within essential genes.

### Tn-seq on pooled sequencing reads demonstrates a GBS A909 essential genome consistent with those of close relatives

Satisfied that data from our three mutant libraries were comparable, we pooled the sequencing reads for each time point from our pilot and second experiments. We did this in order to maximize the measured saturation of our “master” mutant library, thereby minimizing the number of “false-positive” essential genes in our analysis. Our master library had saturation values between 39 and 46 %, with uniform transposon distribution throughout the genome (Fig. 2). At T_0_, we detected 167,684 unique transposon insertions in our master library. By T_2_, this number had dropped to 136,703 (see Additional file 2: Dataset S1).

Pooled reads that had been trimmed of transposon sequence were aligned to the A909 genome, and the subsequent BAM files were used as input for ESSENTIALS [25]. For each time point, genes with log_2_ FC below the local minimum on the corresponding density vs. log_2_ FC plot (generated from a kernel density function of actual vs. expected transposon insertions per gene) were considered essential. Final gene fitness assignments were made as follows: genes that were essential at all three time points are considered essential; genes that were essential at T_1_ and T_2_ but nonessential at T_0_ are considered critical; genes for which essentiality at any time point could not be determined or which otherwise do not meet one of the above criteria are considered undefined. Using these definitions, 13.5 % of the A909 genes are essential, 1.2 % are critical, and 3.1 % are undefined (undefined genes are generally very short and/or are within a local region of low transposon insertion density, such that an expected insertion rate for that gene is not calculable). The remaining 82.2 % of genes are nonessential.

**Fig. 3:**
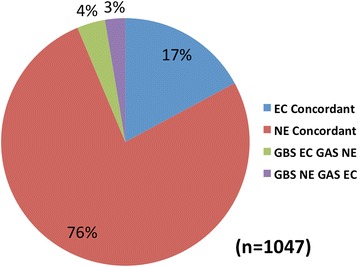
Concordance between GBS and GAS essential genes. Fitness was compared between 1047 orthologous genes of *S. pyogenes* M1T1 5448 and GBS A909. Orthologs were classified as either essential/critical (EC) concordant, nonessential (NE) concordant, or EC in one species and NE in the other

We sought to validate our results with independent Tn-seq fitness data from a closely related but distinct species by comparing our essential and critical genes with those reported for *S. pyogenes* M1T1 5448 by Le Breton et al. [23]. We identified orthologous gene pairs between GBS A909 and *S. pyogenes* strain MGAS5005, which Le Breton et al. used as the reference genome for M1T1 5448 fitness assignments. Fitness comparisons between the two Tn-seq datasets demonstrated close agreement (Fig. 3). Given that Le Breton’s group used a different transposon delivery system, analysis software package [26], and species, and that their data set was similarly validated against Tn-seq data from *Streptococcus sanguinis* and *Streptococcus pneumonia*e, we conclude that the concordance between our fitness determinations attests to the validity of both.

### A909 essential and critical genes map to fundamental KEGG pathways and signaling pathways that control essential and critical genes

We used the gene set enrichment analysis function on the Genome2D webserver [27] to identify A909 KEGG pathways that were enriched for essential and critical genes. The pathways identified serve fundamental cellular housekeeping roles, such as aminoacyl-tRNA synthesis, glycolysis, and nucleotide metabolism (Table 1). We mapped our fitness data onto regulons predicted by RegPrecise to be active in A909 based on the presence of sequences that encode known transcription factors, transcription factor binding sites, or RNA-based signal transduction mechanisms [28]. We also assessed the fitness of two-component system genes predicted by P2CS [29] (Additional file 3: Dataset S2).

**Table 1:** Top KEGG pathways enriched with essential/critical genes

KEGG Class	Essential/Critical Genes	Description
970	33	Aminoacyl-tRNA biosynthesis
240	43	Pyrimidine metabolism
230	47	Purine metabolism
10	40	Glycolysis/Gluconeogenesis
550	13	Peptidoglycan biosynthesis

**Fig. 4:**
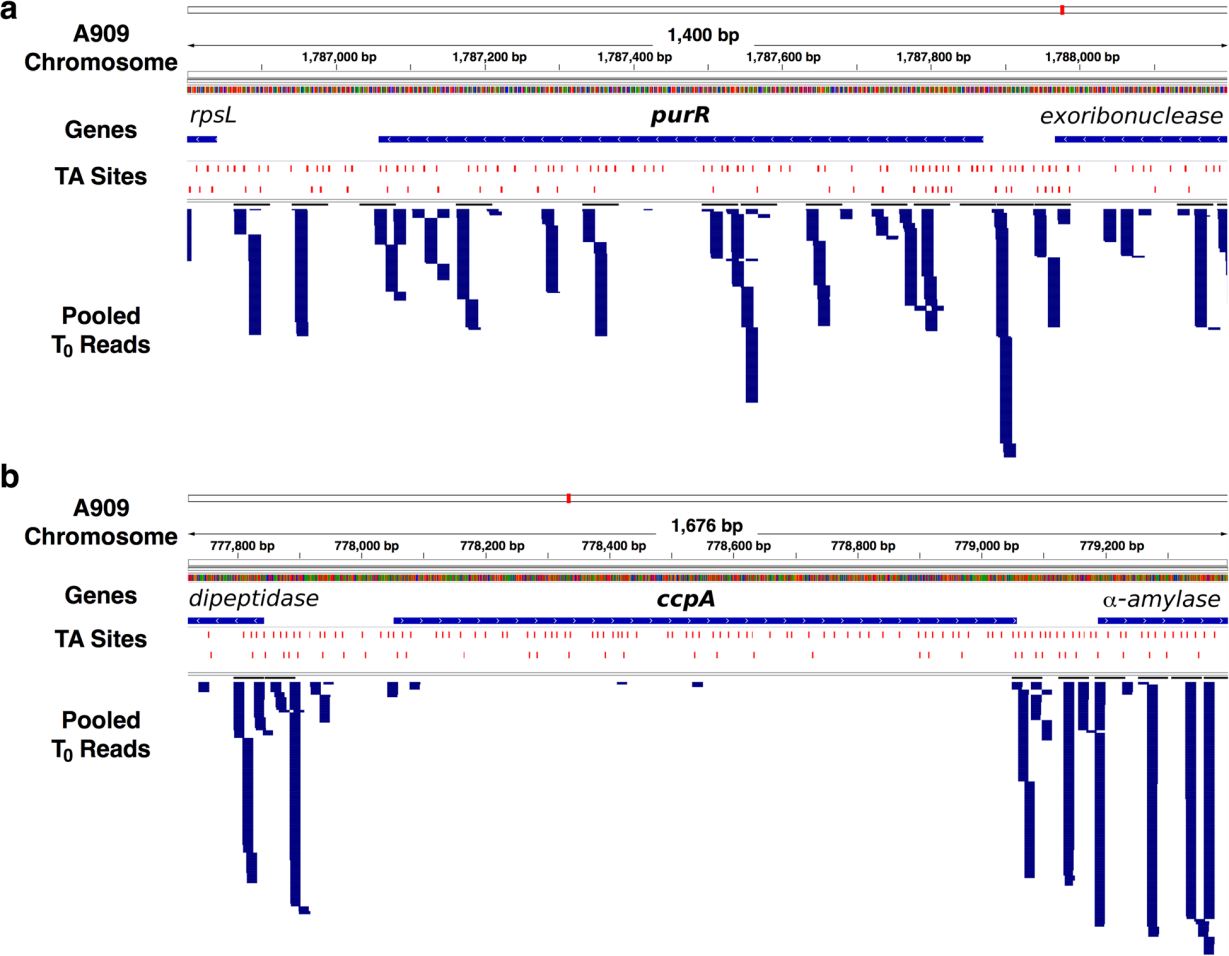
Examples of Tn-seq reads mapped onto essential and non-sessential genes. Pooled T_0_ reads mapped onto transcriptional regulator genes demonstrating characteristic insertion patterns of nonessential genes, such as *purR* (**a**), and essential genes, such as *ccpA* (**b**). For each panel, the rows (from top to bottom) denote the visualized site on the A909 chromosome (with nucleotide number labeling), the gene of interest and its flanking neighbors, available TA sites for transposon insertion, and locations of mapped reads

**Fig. 5:**
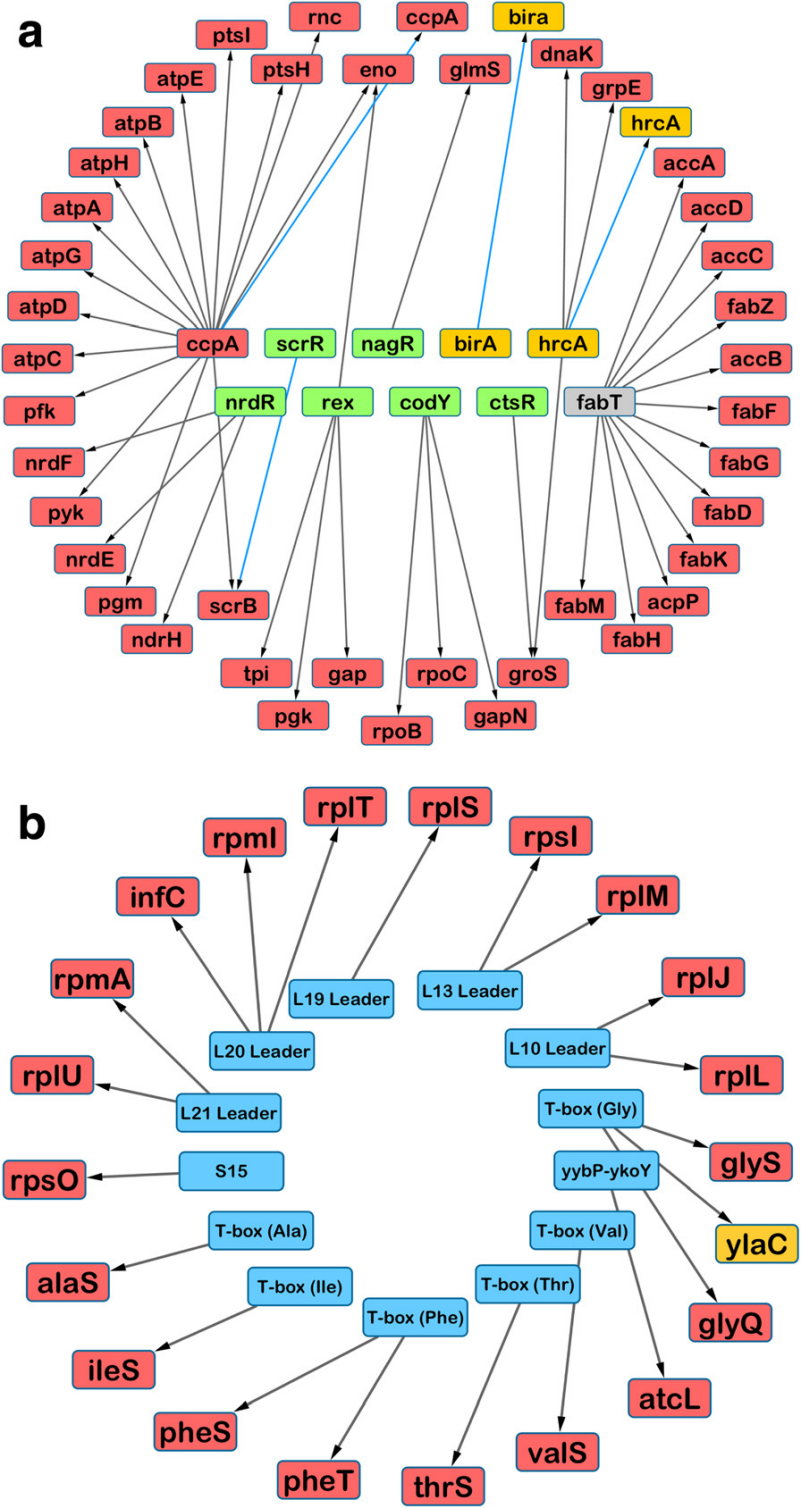
A909 fitness data mapped onto transcription factor- and RNA-based regulons. Essential and critical genes involved in transcription factor- (**a**) and RNA-based (**b**) regulons are depicted. The center set consists of regulatory genes or RNA regulators predicted to affect transcription of genes in the outer ring. Individual genes are color-coded (green = nonessential, yellow = critical, red = essential, gray = undefined). Transcription factor autoregulatory signaling is denoted with a blue arrow

**Fig. 6:**
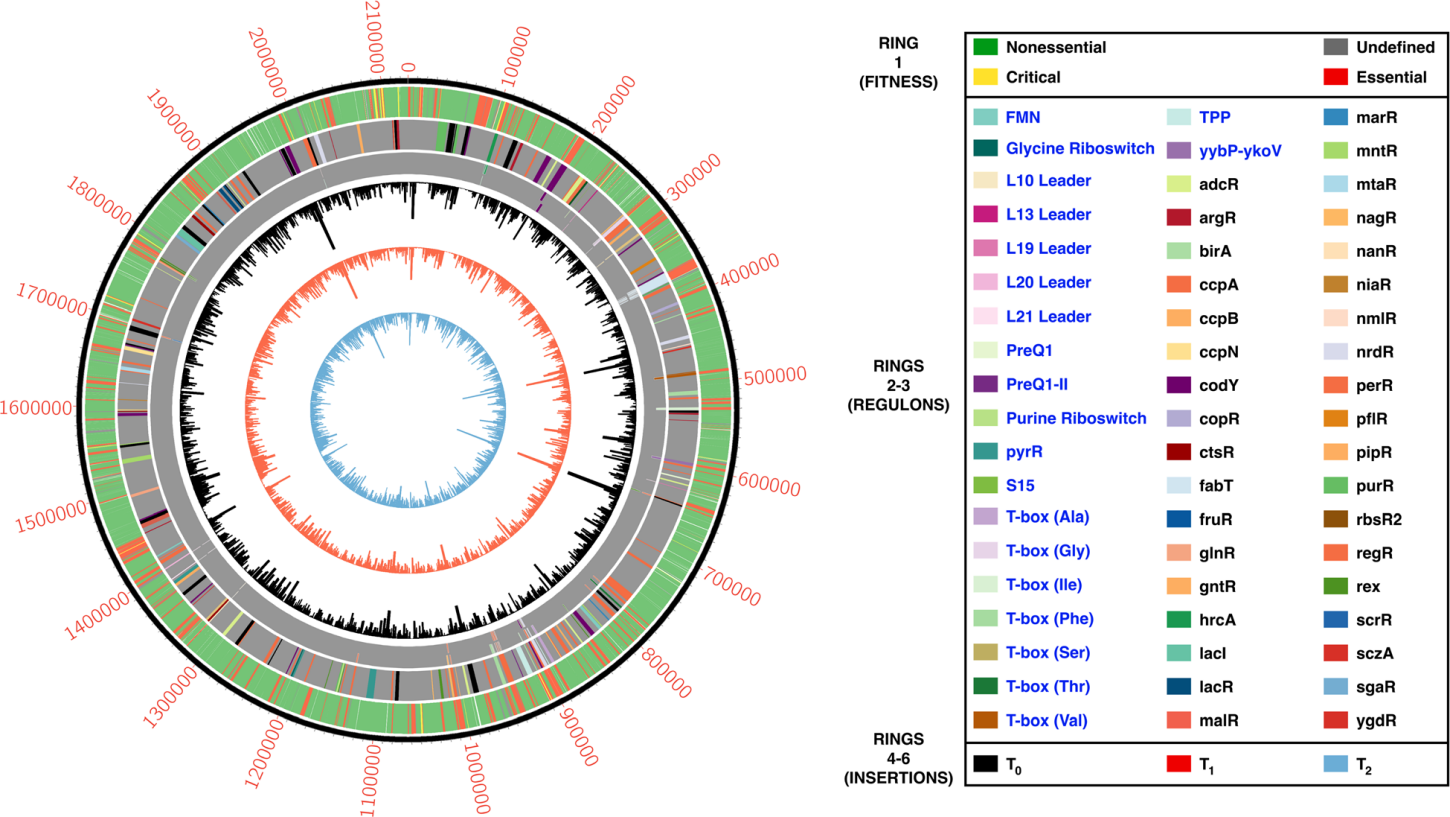
Circos plot of gene fitness, regulons, and library insertion metrics. The outer ring (ring 1) illustrates gene fitness categorizations from consensus data generated by all pooled libraries and time points. The next concentric ring (ring 2) shows all genes that are predicted to participate in an RNA-based (blue text in legend) or transcription factor-based (black text in legend) regulon. Genes that participate in multiple regulons are black. Essential or critical genes that participate in a regulon are tiled in the next ring (ring 3; if an essential or critical gene participates in multiple regulons, the appropriately colored tiles are stacked). The inner three rings (4–6) show unique transposon insertions per gene for each of the three pooled time points

Figure 4 illustrates representative examples of essential and nonessential genes involved in signal transduction. Figure 5 highlights transcription factor- and RNA-based predicted regulons wherein either the regulator, the gene target, or both are either essential or critical based on our data. Interestingly, the regulons diagrammed include both intracellular and cell-wall associated (such as *pgk*) genes [30]. Some signal transduction genes that are dispensable in other Gram-positive bacteria, such as the catabolite control protein gene *ccpA*, are essential in A909 [31, 32], while others that have proven essential in other bacteria—such as the global transcriptional regulator gene *codY*—are nonessential in A909 [33]. Figure 6 summarizes fitness assignments, gene participation in signaling pathways, and library metric data in a single Circos plot [34].

## Discussion

We have developed and validated a system for performing Tn-seq in GBS. Functional analysis of a specific bacterial gene often depends on the ability to generate a knockout (and complement the mutation). However, a significant percentage of bacterial genes are essential for the cell’s survival [35], hence unapproachable using traditional knockout techniques. Until now, resources to make an *a priori* determination of a GBS gene’s fitness have been limited. Jones et al. used signature-tagged mutagenesis in a rat sepsis model to identify GBS genes essential for virulence [36]. While informative, this approach—which employed inocula consisting of 80 pooled transposon mutants—does not permit high-resolution, genome-wide fitness scores. By harnessing the power of NGS to analyze transposon insertion frequency from a saturated and unbiased mutant library, Tn-seq overcomes this limitation. Our baseline fitness data should prove valuable to GBS researchers attempting to generate new knockout strains. These data also offer insights into basic GBS cell biology, delineating key pathways whose presence is necessary for in vitro survival and growth.

Tn-seq data can be superimposed onto novel or existing models of cellular processes, identifying essential or critical nodes in bacterial molecular networks. We have provided examples of such modeling, overlaying A909 gene fitness data onto predicted GBS signaling pathways. We were interested to learn from this analysis that CcpA is predicted to control multiple essential genes, and that the *ccpA* gene is itself essential (see Fig. 4). This finding aligns with our experience of unsuccessfully attempting to generate a *ccpA* knockout in multiple GBS strains (unpublished). However, other broadly active transcription factors, such as *codY,* are nonessential. The fatty acid biosynthesis regulator FabT is notable for its control over the completely essential *fab* operon, also referenced in previous Tn-seq studies [23]. In our strain, the fitness of the *fabT* gene itself (A909 locus SAK_0417) is undefined, due to its short length (435 bp) and its position in a region of low transposon insertion density.

Our three mutant libraries showed reproducible insertion distributions (see Fig. 2). Overall, there was widespread and uniform dispersion of mini-transposon insertions throughout the genome. Regions of low insertion density (corresponding to essential genes) were consistent across our biological replicates, generating the clustered points near the origins of the inter-library correlation plots in Fig. 2d. As expected, we noted consistently low insertion density in regions of the chromosome rich in ribosomal protein coding sequences (approximately gene loci 20–50 and 90–120 in Fig. 2a). Key housekeeping genes, such as *dnaA* and *dnaE* were consistently uninterrupted, as expected.

Even with pooled sequencing reads, we did not achieve levels of transposon saturation as high as reported in some previous studies; however, saturation levels range widely between different Tn-seq studies, and the saturation of our pooled reads (ranging from 39 to 45 %) is well above that of mutant libraries used for Tn-seq in other species [37]. The key measure for making fitness determinations through Tn-seq is not the absolute saturation of the library, but whether nonessential genes are consistently and uniformly interrupted by transposon insertions. In our master T_0_ library, 98.8 % of genes aligned to at least one mapped read, and 99.5 % of nonessential genes were interrupted at between three and 783 unique TA sites. In the case of essential and critical genes, any reads that aligned tended to be very low frequency and clustered at the 5’ and 3’ ends of the gene (for example, see the *ccpA* gene in Fig. 4b).

One potential application of this system is toward identification of drug and/or vaccine targets that impair GBS growth. Multiple candidate GBS vaccines against capsular polysaccharides and cell-wall associated proteins have been developed, and some are still undergoing testing [38–41], but efforts in this area have been hindered by serotype specificity and incomplete immunity. Tn-seq fitness data presented in this work might aid in this work by identifying potential antibody targets whose inactivation by binding would impair essential pathways. Similarly, small molecule inhibitors of bacterial intracellular signaling and/or metabolic pathways have been proposed as novel antibiotics or adjuvants [42–44]. The search for novel drug targets may benefit from access to fitness data such as ours.

A major advantage of Tn-seq over traditional mutant library screening is the flexibility it offers in experimental design. In vitro work such as we report here can be complemented and enhanced by performing Tn-seq on libraries grown under biologically challenging conditions, such as in human fluids or in vivo [24, 37, 45, 46], establishing conditionally essential genes for a given growth environment [25, 45, 47]. Our system should be easily transferable to other, more physiologically relevant experimental conditions. Work toward extending our findings to other GBS strains and growth conditions is underway.

## Conclusions

We have developed and tested a reliable system for performing Tn-seq in GBS, and have determined the essential genome of strain A909. This Tn-seq system is flexible and should permit assessment of conditionally essential genes from biological challenge experiments (including in vivo systems).

## Methods

### Bacterial strains and growth conditions

GBS strain A909—a serotype Ia, sequence type 7 (ST 7) GBS strain widely used in laboratory investigations—and its derivatives were grown stationary in TS media (Fisher Scientific product number DF0370-17-3) at 28 or 37 °C, supplemented with Erm 5 μg/mL and/or Km 1000 μg/mL as needed for selection. *E. coli* were maintained in Luria broth (LB) media at 28 or 37 °C, supplemented with Erm 200 μg/mL and/or Km 50 μg/mL as needed for selection. pCAM45 was maintained in *E. coli* strain JM109. Cloning steps to develop pCAM48 were performed in *E. coli* strain DH10B.

### Construction of pCAM48

pCAM45 was used as PCR template for Gibson assembly of a 6677-bp vector fragment amplified using primers pCAM46 GA F and pCAM46 GA R and a 1475-bp mini-transposon fragment modified with point mutations to contain MmeI restriction enzyme sites in its terminal inverted repeats, which was amplified using primers Himar1 GA F and Himar1 GA R (see Additional file 4: Dataset S3). Subsequent Sanger sequencing of plasmid DNA from pCAM46 confirmed the presence of the MmeI sites, and digestion of the plasmid with MmeI generated the expected 1463-bp transposon band, but the second band, representing linearized vector, was approximately 1500 bp smaller than expected, suggesting that there had been a partial deletion of the vector outside of the mini-transposon region. To correct this, pCAM45 was linearized with PvuI, which excised the non-mutagenized mini-transposon region. The corresponding mini-transposon region from pCAM46 was also excised with PvuI. The two fragments were ligated to generate pCAM47, which was subsequently confirmed to generate fragments of the expected size after digestion with BamHI, MmeI, and AvaI. Finally, to correct the point mutation in the pCAM47 temperature-sensitive origin of replication (described by May et al. [20]), that region was excised from pCAM47 by double-digest with BamHI and XbaI. The correct sequence was amplified from pHY304 using primers TS ORI F and TS ORI R, and the two fragments were Gibson assembled.

### A909 transformation with pCAM48

Competent A909 stocks were prepared based on methods described by Holo and Ness [48]. Starter cultures were used to seed 5 mL preparations of M17 broth with 0.5 % glucose. After overnight growth, this culture was used to seed 100 mL preparations of M17 broth with 0.5 % glucose, 0.5 M sucrose, and 2.5 % glycine. After overnight growth, these cultures were spun down and washed twice in an aqueous solution of 0.5 M sucrose and 10 % glycerol. After a third spin, the bacteria were resuspended in 1 mL of wash solution and stored in 50 μL aliquots at −80 °C until transformation. To transform with pCAM48, 5 μL of purified plasmid was added to a 50 μL competent A909 aliquot on ice. The mixture was transferred to a prechilled 0.2 cm gap electroporation cuvette and pulsed once with 2.5 kV (25 μF, 200 Ω). After addition of 500 μL ice cold M17 broth with 0.5 % glucose, 0.5 M sucrose, 20 mM MgCl_2_, and 2 mM CaCl_2_, the mixture was kept on ice for 5 min, then transferred to 28 °C for 2 h. After outgrowth, serial dilutions were plated on TS plates with Km at 28 °C.

### Generation of A909 mutant libraries

Mutant libraries were generated using methods adapted from Le Breton and McIver [49] and van Opijnen et al. [3]. To verify the expected phenotype of pCAM48 transformants, individual colonies of A909 transformed with pCAM48 were patched to three selection conditions: 1) TS Km + Erm at 28 °C; 2) TS Erm at 37 °C; 3) TS Km at 37 °C. Clones that grew in conditions 1 and 2 but not 3 were scraped from the Km + Erm 28 °C plates and used to generate glycerol stock libraries. Eight out of 23 patched colonies demonstrated the correct antibiotic resistance phenotype and were stocked. These suspensions were then serially diluted and plated on TS Km + Erm at 28 °C and TS Erm at 37 °C. Colony counts from the two conditions were used to establish transposon insertion frequency in the stocked libraries.

For each candidate library, 100 individual colonies from the TS Erm 37 °C plate, assumed to have undergone genomic transposon insertion, were patched to TS Km plates at 37 °C and 20 individual colonies were used to seed liquid TS Erm cultures at 37 °C. The TS Km 37 °C plates were used to establish the rate of non-productive vector insertion into transposon insertion sites (i.e. to select for clones in which homologous recombination between a chromosomal transposon insertion and pCAM48 had occurred); libraries with a rate >5 % were discarded. Five libraries with non-productive integration rates between 6.7 % and 80 % were discarded. Genomic DNA extracted from the 20 TS Erm 37 °C liquid cultures was used as template for arbitrary priming APPCR using PCR primers APPCR Tn F and APPCR DegTail R in the first reaction, then APPCR Tn F and APPCR R in the second reaction [49]. Purified DNA from the second reaction was Sanger sequenced using APPCR Anchor as the sequencing primer. Libraries with >5 % identical transposon insertion sites (i.e. one pair of siblings among 20 tested clones) were planned to be discarded, but no siblings were observed among the tested samples (one sequencing reaction did not work). Finally, to improve curing of pCAM48, aliquots of the stock samples were plated onto 80 150x15 mm TS Erm plates at 37 °C. After 48 h of growth, colonies were scraped off and used to generate final glycerol library stocks for use in Tn-seq.

### Library growth, DNA preparation, and sequencing

Five milliliter of final library stocks were used to seed 250 mL liquid TS Erm cultures at 37 °C, while 10 mL of the library stocks were serially washed with PBS to remove glycerol, then used for T_0_ genomic DNA extraction. After 24 h of growth, a 30 mL aliquot of the liquid culture was used for T_1_ DNA extraction, while 10 mL was used to seed another 250 mL TS Erm culture at 37 °C. The process was repeated for the remaining time points. DNA extractions were performed on pelleted bacterial samples resuspended in 150 μL PBS using the MoBio Powersoil kit according to manufacturer instructions. Following extraction, the genomic DNA samples were digested with MmeI, then ligated overnight to barcoded adapters prepared as previously described [3]. Selective PCR amplification of transposon-genome junctions was performed using primers Illumina PCR Tn F and Illumina PCR Adapt R. PCR was limited to 20–26 cycles in order to remain in the linear phase of template amplification. Following PCR and agarose gel electrophoresis, the expected 189-bp band was excised and gel extracted using the Qiagen QIAquick kit. See Additional file 5: Dataset S4 for details about the expected PCR product. Purified samples were assessed on an Agilent Bioanalyzer before sequencing. Amplicon samples were multiplexed and sequenced on a 150 nt paired-end run of the Illumina HiSeq 4000 platform with a target number of reads per library of ~15 M. Demultiplexing and read binning was performed using the open source tool FastqMultx (https://code.google.com/p/ea-utils/wiki/FastqMultx).

### Determination of the GBS essential genome

Demultiplexed and trimmed Illumina reads were initially visualized using Geneious. Library statistics are included in the Additional file 2: Dataset S1. Reads were aligned to the expected Tn-seq 189-bp product in order to isolate the internal 17-bp GBS genomic sequences. These were extracted to a new list and aligned to the A909 genome using Bowtie2, generating SAM and BAM files. The SAM file was used as input for Tn-seq Explorer [50], which was used to generate transposon insertion metrics, and for visualization in Integrated Genomics Viewer (IGV) [51]. The following statistical analysis was performed in order to establish that transposon insertion was unbiased. For each library at T_0_, the genome was divided into 100 equally sized segments, and the number of unique insertions determined for each segment. The Kolmogorov-Smirnov (K-S) test of normality was then used to establish whether the distribution of insertion counts in the segments was skewed in any of our libraries. Such skew, if present, would lead to rejection of the null hypothesis that the counts were normally distributed, and would suggest a predilection for transposon insertion in one portion of the genome over another. K-S p values were >0.2 for all libraries, indicating that the insertions were evenly distributed at a genomic level. The BAM file was used as input for ESSENTIALS [25], with the following settings: minimum sequence match length of 12 bp; permitting alignment to both the forward and reverse strands; repeat filtering on; LOESS correction to remove genomic position bias; TMM for read count normalization; tagwise modeling of variance with amount of smoothing set to 5. The expected vs. experimental insertion density fold-change cutoff generated by ESSENTIALS was used to separate essential from nonessential genes for that library/time point pair.

### Comparison with *S. pyogenes* Tn-seq data

Supplemental tables from Le Breton et al. [23] were downloaded and parsed to extract fitness assignments for *S. pyogenes* strain M1T1 5448. Orthologs between *S. pyogenes* strain MGAS5005 (which Le Breton et al. used as the reference genome for M1T1 5448) and A909 were determined using the Microbial Genome Database for Comparative Analysis (MBGD) [52]. Orthologous pairs that were essential, critical, or nonessential in one or both of the analyses were compared to produce consensus data.

### Mapping of essential/critical genes onto expected KEGG and signaling pathways

After determination of essential/critical genes from ESSENTIALS analysis, KEGG pathway enrichment was assessed using the gene set enrichment analysis on the Genome2D webserver (http://server.molgenrug.nl) [27]. Putative A909 regulons were determined from the RegPrecise analysis of the GBS 2603 V/R reference strain [28]. Orthologs between 2603 V/R and A909 were determined using MBGD. Putative A909 two-component systems were obtained from the P2CS webserver (http://www.p2cs.org) [15, 29].

## Abbreviations

APPCR, arbitrary priming; FC, fold-change; GBS, group B *Streptococcus*; Km, kanamycin; K-S, Kolmogorov-Smirnov; NGS, next-generation sequencing; PCR; Erm, erythromycin; Tn-seq, sequencing of transposon-genome junctions; TS, tryptic soy.

## Additional files

Additional file 1: Figure S1. Arbitrary Priming PCR Shows Randomly Distributed Transposon Insertions Among 19 Mutant Clones and Identifies a *cylE* Insertional Knockout. Geneious alignment image from 19 Sanger sequencing reactions performed from APPCR on individual mutant A2 library clones, showing random distribution along the A909 genome (green bar at bottom) and no siblings with identical insertions (A). Expected orientation of transposon in the *cylE* insertional knockout, with confirmatory PCR primer locations and agarose gel of confirmatory PCR (B). Pigment phenotype of WT A909 and *cylE* insertional knockout grown in new Granada media (C). (PDF 1154 kb) Additional file 2: Dataset S1. Mutant Library Sequencing Metrics. Illumina sequencing data are listed for each of three mutant libraries (A2, A5, and A7) at 3–4 time points (A2 library DNA was collected at four time points during the pilot experiment; the remaining experiments used three time points). Data for pooled experimental reads are also provided. Total reads per condition are listed in column B. Column C lists the percent of reads whose genome junction sequence aligned to the A909 genome. Column D lists the percent of non-GBS aligning reads that aligned to pCAM48 (indicating low-level persistence of the plasmid in the library population). Column E indicates the percent of total reads represented by the values in column D. Column F indicates percent of non-GBS aligning reads that did not align to pCAM48. Column G lists the total number of unique TA sites bearing a transposon insertion in each condition. (PDF 20 kb) Additional file 3: Dataset S2. Detailed Gene Fitness Data. Tab 1 (A909 Final Fitness Assignments) lists A909 genes alongside fitness classification as outlined in the main text. Tabs 2–4 (T0-3 ESSENTIALS Output) show raw data from ESSENTIALS analysis of sequencing data from pooled reads at each of three time points. The columns list (from left to right) gene loci, TA sites per locus, the expected insertion number given the null hypothesis that the gene is nonessential, the log_2_ FC value for that gene’s experimental vs. expected insertion count, the p value for the FC (generalized linear model likelihood ratio test, corrected for multiple comparisons), start and stop base pair positions for the gene’s ORF, the strand on which the ORF is read, the gene length, its name (if named), and the protein product (if known). Tab 5 (RegPrecise Regulons) lists predicted GBS regulon gene participants. Gene loci are provided for the GBS reference strain 2603 V/R and the A909 homolog. Final fitness category assignments are provided for each gene. Tab 6 (2-Component Systems) shows predicted gene participation in two-component systems identified by P2CS. Columns (from left to right) list GBS gene loci, the original literature description of the gene product function (not necessarily in GBS), the gene’s fitness category assignment, the functional class predicted by domain architecture (either response regulator [RR] or sensor histidine kinase [HK]), and whether the function of the gene or its homolog has been validated experimentally. (XLSX 815 kb) Additional file 4: Dataset S3. Primers and Plasmids Used in this Study. (PDF 25 kb) Additional file 5: Dataset S4. Amplicon Details. Nucleotide sequence and barcode information for the amplicons generated in this study. (PDF 32 kb)
