# Supplementary figures and images for: The essential genome of Streptococcus agalactiae
Source: BMC Genomics. 2016 May 26;17:406. doi: 10.1186/s12864-016-2741-z (PMC4881062; doi:10.1186/s12864-016-2741-z)

A.

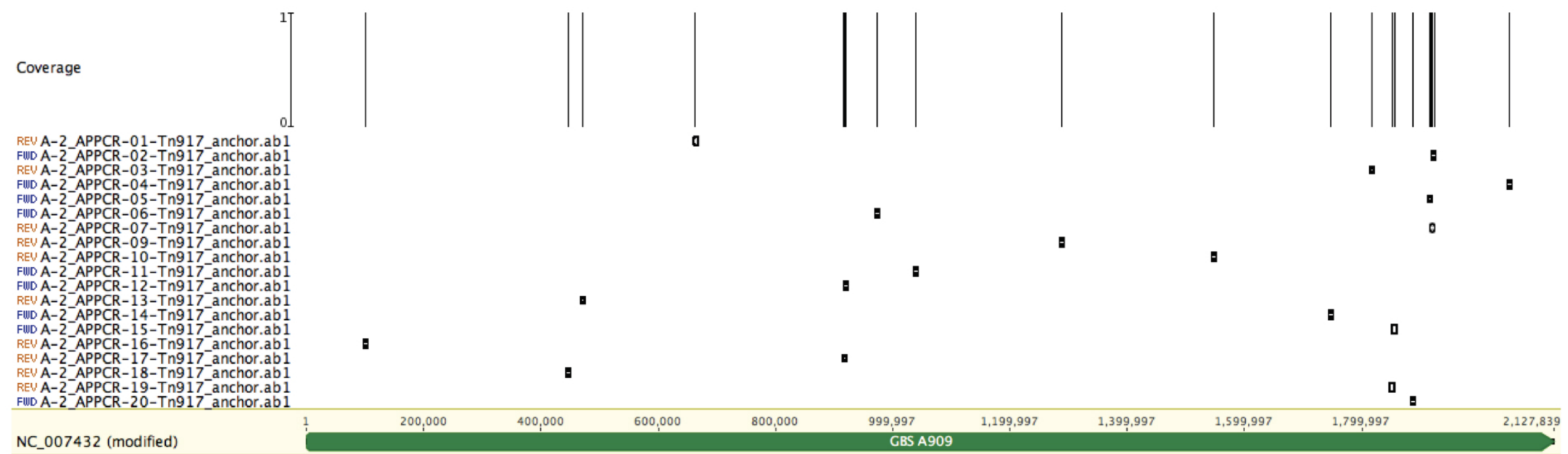

B.

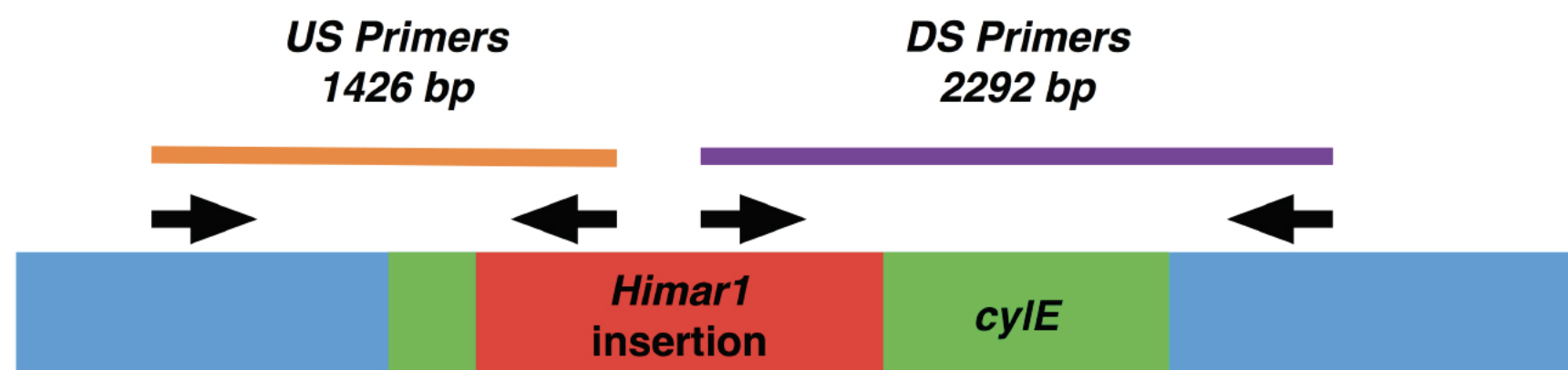

3000 bp  
2000 bp  
1500 bp  
1000 bp  
500 bp

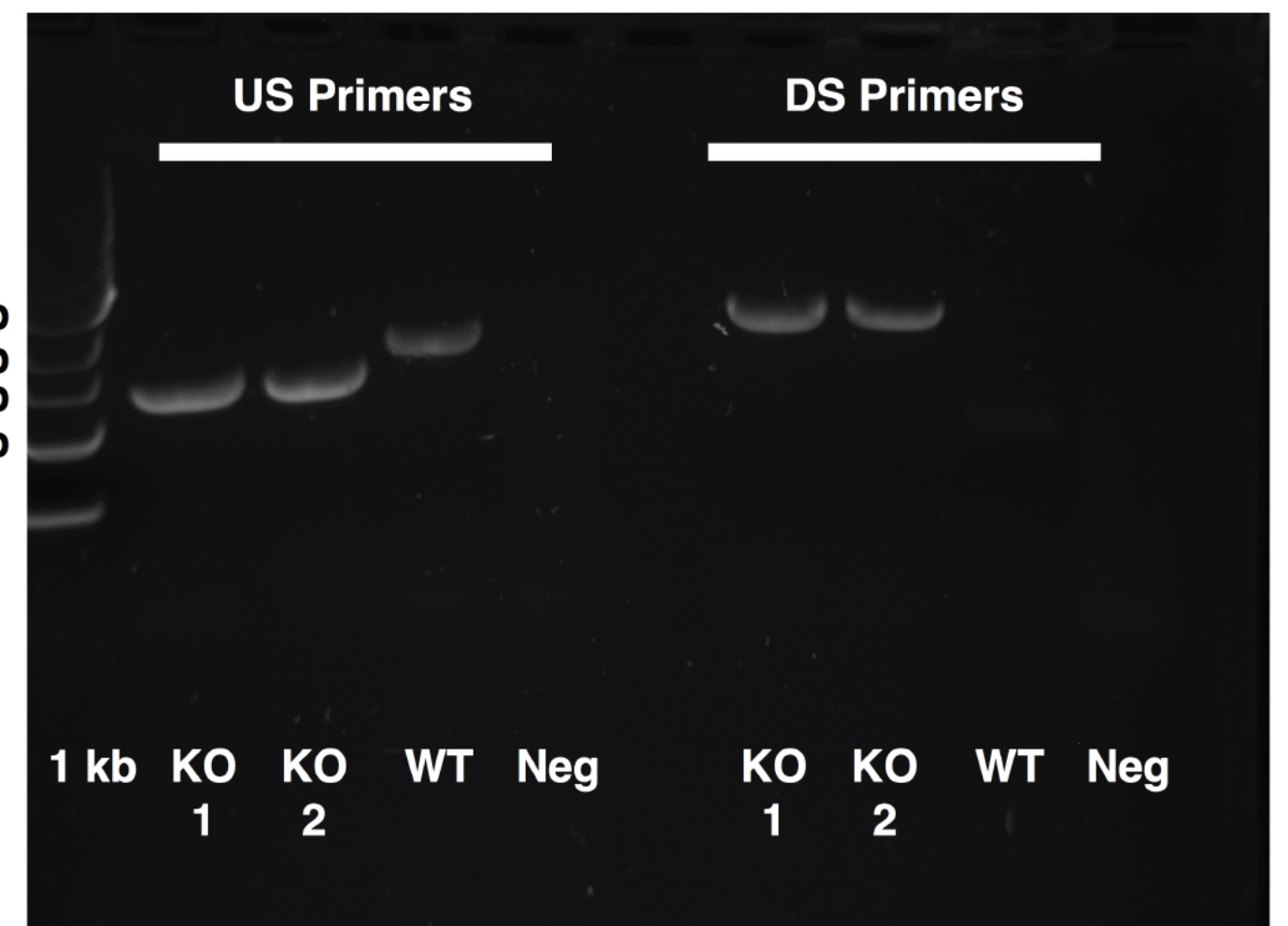

C.

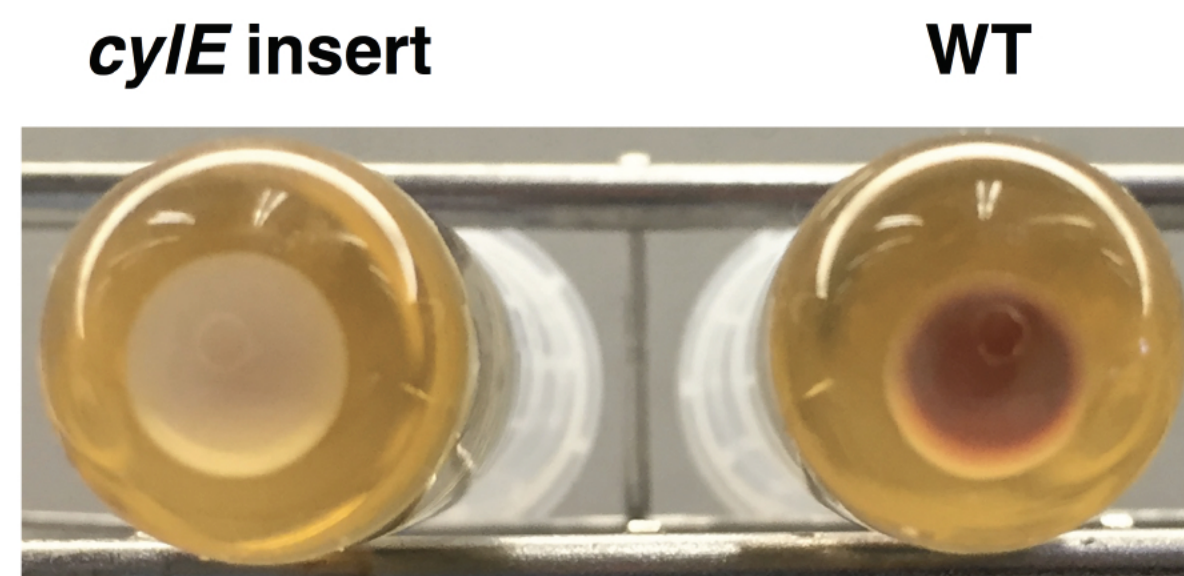

Supplement: Additional file 1: Figure S1. — Arbitrary Priming PCR Shows Randomly Distributed Transposon Insertions Among 19 Mutant Clones and Identifies a cylE Insertional Knockout. Geneious alignment image from 19 Sanger sequencing reactions performed from APPCR on individual mutant A2 library clones, showing random distribution along the A909 genome (green bar at bottom) and no siblings with identical insertions (A). Expected orientation of transposon in the cylE insertional knockout, with confirmatory PCR primer locations and agarose gel of confirmatory PCR (B). Pigment phenotype of WT A909 and cylE insertional knockout grown in new Granada media (C). (PDF 1154 kb) [file 12864_2016_2741_MOESM1_ESM.pdf]
