# Supplementary material for: The essential genome of Streptococcus agalactiae
Source: BMC Genomics. 2016 May 26;17:406. doi: 10.1186/s12864-016-2741-z (PMC4881062; doi:10.1186/s12864-016-2741-z)
Supplement: Additional file 2: — Dataset S1. Mutant Library Sequencing Metrics. Illumina sequencing data are listed for each of three mutant libraries (A2, A5, and A7) at 3–4 time points (A2 library DNA was collected at four time points during the pilot experiment; the remaining experiments used three time points). Data for pooled experimental reads are also provided. Total reads per condition are listed in column B. Column C lists the percent of reads whose genome junction sequence aligned to the A909 genome. Column D lists the percent of non-GBS aligning reads that aligned to pCAM48 (indicating low-level persistence of the plasmid in the library population). Column E indicates the percent of total reads represented by the values in column D. Column F indicates percent of non-GBS aligning reads that did not align to pCAM48. Column G lists the total number of unique TA sites bearing a transposon insertion in each condition. (PDF 20 kb) [file 12864_2016_2741_MOESM2_ESM.pdf]

Library	Total paired-end reads	Percent of processed reads aligned to A909	Percent of non-mapping reads that align to pCAM48	Percent of total reads that align to pCAM48	Percent of non-mapping reads that are erroneous (non-aligning)	Unique TA site insertions
A2 pilot T0	8,244,964	87.9	99.9	12.1	0.1	19,716
A2 pilot T1	8,919,568	91.0	99.8	9.0	0.2	18,807
A2 pilot T2	11,632,546	89.0	99.5	10.9	0.5	25,847
A2 pilot T3	8,520,362	94.2	99.8	5.8	0.2	11,954
A2 repeat T0	44,760,116	87.1	82.3	10.6	17.7	36,030
A2 repeat T1	30,443,676	84.8	11.6	1.8	88.4	31,044
A2 repeat T2	23,541,808	82.7	39.9	6.9	60.1	25,631
A5 repeat T0	20,790,076	82.9	90.1	15.4	9.9	100,800
A5 repeat T1	20,433,314	80.7	27.2	5.2	72.8	83,730
A5 repeat T2	31,774,390	83.7	39.6	6.5	60.4	89,716
A7 repeat T0	14,373,542	88.8	83.4	9.3	16.6	68,615
A7 repeat T1	14,375,780	88.3	49.7	5.8	50.3	52,250
A7 repeat T2	17,445,696	73.1	24.8	6.7	75.2	47,084
Pooled T0	88,168,698	86.4	86.3	11.7	13.7	167,684
Pooled T1	74,172,338	85.1	40.3	6.0	59.7	139,623
Pooled T2	84,394,440	82.0	40.2	7.2	59.8	136,703
