# Supplementary material for: The essential genome of Streptococcus agalactiae
Source: BMC Genomics. 2016 May 26;17:406. doi: 10.1186/s12864-016-2741-z (PMC4881062; doi:10.1186/s12864-016-2741-z)
Supplement: Additional file 4: — Dataset S3. Primers and Plasmids Used in this Study. (PDF 25 kb) [file 12864_2016_2741_MOESM4_ESM.pdf]

Plasmid Name	Description	Reference	GenBank Accession
pCAM45	*Himar1* Gram-positive transposon mutagenesis vector with temperature-sensitive origin of replication (non-functional due to point mutation)	May, J. P., Walker, C. A., Maskell, D. J. & Slater, J. D. Development of an in vivo Himar1 transposon mutagenesis system for use in Streptococcus equi subsp. equi. FEMS Microbiol. Lett. 238, 401–409 (2004).	AY680862.1
pCAM46	pCAM45 modified so that *Himar1* mini-transposon has MmeI sites in inverted repeat regions; wrong size due to unspecified internal deletion	This study	
pCAM47	*Himar1* mini-transposon with MmeI sites excised from pCAM46 and ligated into pCAM45 to repair internal deletion	This study	
pCAM48	pCAM47 with temperature-sensitive origin of replication replaced with correct sequence amplified from pHY304	This study	KU936423
pHY304	Shuttle vector with temperature-sensitive origin of replication, used as template for generation of pCAM48	Yim, H. H. & Rubens, C. E. Site-specific homologous recombination mutagenesis in group B streptococci. Methods in cell science 20, 13–20 (1998).	KU936424
