# Supplementary material for: The essential genome of Streptococcus agalactiae
Source: BMC Genomics. 2016 May 26;17:406. doi: 10.1186/s12864-016-2741-z (PMC4881062; doi:10.1186/s12864-016-2741-z)
Supplement: Additional file 5: — Dataset S4. Amplicon Details. Nucleotide sequence and barcode information for the amplicons generated in this study. (PDF 32 kb) [file 12864_2016_2741_MOESM5_ESM.pdf]

*S. agalactiae* A909 TnSeq Supporting Documentation

Expected fragment characteristics:

189 bp

AATGATACGGCGACCACCGAGATCACACTCTTTCCCTACACGACGCTCTTCCGATCT*XX*
*XXXXXX***NNNNNNNNNNNNNNTA**ACAGGTTGGATGATAAGTCCCCGGTCTGACACATAGA
TGGCGTCGCTAGTATTAAATGCAGTAGATCCGAAGATCAGCAGTTCAACCTCGTATGCC
GTCTTCTGCTTG

Single Underline: Illumina-specific sequence
*XXX*: Barcode
**Bold:** GBS sequence (from strain A909)
Double Underline: MmeI binding site

Mutant Libraries:
A2
A5
A7

Each mutant library prep has time point-specific barcodes:
T0: TATAGCCT
T1: ATAGAGGC
T2: CCTATCCT
T3 (A2 pilot only): GGCTCTGA
